# Myeloid suppressor cells require membrane TNFR2 expression for suppressive activity

**DOI:** 10.1002/iid3.19

**Published:** 2014-05-28

**Authors:** Johannes Polz, Annika Remke, Sabine Weber, Dominic Schmidt, Dorothea Weber-Steffens, Anne Pietryga-Krieger, Nils Müller, Uwe Ritter, Sven Mostböck, Daniela N Männel

**Affiliations:** Institute of Immunology, University of RegensburgRegensburg, Germany

**Keywords:** Immune suppression, inflammation, tumor necrosis factor

## Abstract

TNF and TNF receptor type 2 (TNFR2) have been shown to be important for generation of myeloid-derived suppressor cells (MDSC). In order to analyze whether and how TNFR2 passes the effect of TNF on, myeloid cells from TNFR2-deficient mice were compared to respective cells from wild-type mice. Primary TNFR2-deficient myeloid cells showed reduced production of NO and IL-6 which was attributable to CD11b^+^ CD11c^−^ Ly6C^+^ Ly6G^−^ immature monocytic MDSC. TNFR2-deficient MDSC isolated from bone marrow were less suppressive for T cell proliferation compared to WT-derived MDSC. These differences on myeloid cells between the two mouse lines were still observed after co-culture of bone marrow cells from the two mouse lines together during myeloid cell differentiation, which demonstrated that the impaired functional capacity of TNFR2-deficient cells was independent of soluble factors but required membrane expression of TNFR2. Similarly, adoptive transfer of TNFR2-deficient bone marrow cells into wild-type hosts did not rescue the TNFR2-specific phenotype of bone marrow-derived myeloid cells. Therefore, membrane TNFR2 expression determines generation and function of monocytic MDSC.

## Introduction

TNF is a key inflammatory cytokine regulating the immune system. TNF is well known to cause inflammatory reactions such as tissue injury in autoimmune diseases mainly by activation of TNF receptor type 1 (TNFR1). Accordingly, blockade of TNF in patients with chronic inflammation and autoimmune diseases is currently being used therapeutically [1]. However, clinical observations in patients after treatment with TNF antagonists indicated that TNF has more complex immune regulatory properties [2]. Animal studies showed that TNF can exert immune suppressive functions such as suppressed T cell signaling after prolonged TNF exposure [3]. The interaction of TNF with TNFR type 2 (TNFR2) seems to play an important role, in particular for the function of regulatory T cells [4,5]. However, not only regulatory T cells but also myeloid-derived suppressor cells (MDSC) seem to be affected by TNF and TNFR2. TNF-dependent immune suppression correlated with development of functional MDSC in a model of chronic inflammation induced by repetitive application of BCG [6]. In this model, splenic MDSC from TNF-deficient mice produced lower amounts of NO, had lower arginase 1 activity, and were less suppressive for T cell proliferation. In addition, differentiation of myeloid splenocytes to mature macrophages and DC was arrested by TNF. Zhao et al. [7] addressed the question concerning the responsible TNF receptor controlling maturation of MDSC in a tumor model. While tumor rejection was shown to be TNF-dependent and associated with accumulation of MDSC, TNFR2-deficient (TNFR2^−/−^) mice failed to induce MDSC development upon tumor transplantation and showed impaired tumor growth. These authors conclude that TNFR2 expression is required for MDSC accumulation during tumor growth and TNFR2 signaling is necessary and sufficient for protection of MDSC from apoptosis. However, these data do not conclusively answer the question whether this effect is due to soluble or membrane TNFR2.

So far, the importance of TNFR2 on MDSC survival and accumulation was shown, but the mechanism of the TNFR2 impact on MDSC functionality has not been clarified. Therefore, we analyzed the functional capacity of myeloid cells from TNFR2^−/−^ mice and compared in vitro differentiation of bone marrow precursors from TNFR2-competent and TNFR2^−/−^ mice with special attention to the immature myeloid subpopulation of monocytic MDSC. We demonstrate that membrane TNFR2 expression and, therefore, presumably TNFR2 signaling is necessary for optimal function of MDSC.

## Results

### Impaired NO production by TNFR2^−/−^ myeloid cells

**Figure 1:**
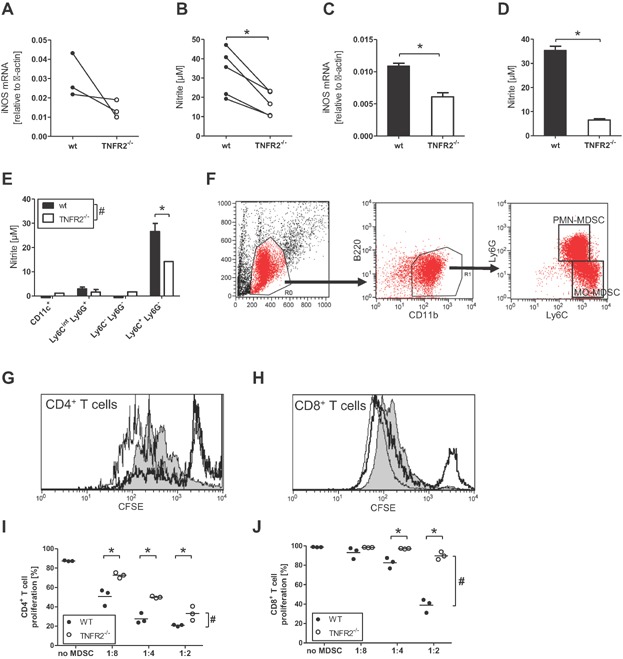
Differences between myeloid cells from WT and TNFR2-defcient mice. (A, B) Following a sterile inflammation by PBS injection, peritoneal exudate cells from WT (black circles) and TNFR2^−/−^ (white circles) mice were activated with LPS plus IFNγ in vitro for 48 h and the levels of (A) iNOS mRNA in cells and (B) nitrite in supernatant were determined. Each symbol represents an independent experiment, * indicates *P* < 0.05 in a paired Student's *t*-test. (C–E) Splenic CD11c^−^ CD11b^+^ myeloid cells were purified from WT (black bars) and TNFR2^−/−^ (white bars) mice 2 days after cecal ligation and puncture (CLP), pooled per group, activated with LPS plus IFNγ in vitro for 48 h and the levels of (C) iNOS mRNA in cells and (D) nitrite in supernatant were determined. Data are a representative of three independent experiments and shown as mean +/− SD of technical replicates, * indicates *P* < 0.05 in Student's *t*-test. (E) Two days after CLP, splenic myeloid cells from WT (black bars) and TNFR2^−/−^ (white bars) mice were pooled per group, sorted into four sub-populations, activated with LPS plus IFNγ in vitro for 48 h and the levels of nitrite in supernatant were determined. Data are from one representative of two independent experiments and shown as mean +/− SD of technical replicates. ^#^ Indicates that two-way ANOVA demonstrated a significant influence (*P* < 0.05) of TNFR2 on the result, * indicates *P* < 0.05 in Bonferroni's post test. (F–J) Two days after CLP, MO-MDSC were enriched from the pooled bone marrows of WT and TNFR2^−/−^ mice by magnetic cell separation and used in a T cell proliferation suppression assay. (F) The gating strategy to identify PMN-MDSC and MO-MDSC with representative data from WT bone marrow on day two after CLP. (G,H) Representative histogram overlays to demonstrate the impact of MO-MDSC on T cell proliferation. Shown are the CFSE profile of T cells activated without added MDSC (gray histogram), with bone marrow MO-MDSC from WT mice (thick black line) or bone marrow MO-MDSC from TNFR2-deficient mice (thin black line). Note the increased population of CFSE-high cells in the cultures with MO-MDSC, indicating reduced proliferation of the T cells. (I,J) Calculation of the proliferative response of CD4^+^ and CD8^+^ T cells in presence of bone marrow MO-MDSC from WT (black circles) or TNFR2-deficient (white circles) mice. Shown is one representative experiment of two independent repeats. Each symbol represents a technical replicate; the horizontal line represents the mean. ^#^ Indicates that two-way ANOVA demonstrated a significant influence (*P* < 0.05) of TNFR2 on the result, * indicates *P* < 0.05 in Bonferroni's post test.

We assayed the function of myeloid cells from multiple tissues of TNFR2^−/−^ mice to elucidate the functional impact of TNFR2. To recruit myeloid cells from the blood into the peritoneum we used a sterile inflammation by PBS injection to elicit peritoneal exudate cells (PEC) consisting to >50% of macrophages as determined by morphology. Such PEC of TNFR2^−/−^mice responded with reduced iNOS mRNA expression and lower NO production to stimulation with LPS plus IFNγ when compared to PEC from wild-type (WT) mice (Fig. 1A,B). To activate myeloid cells in vivo for effective NO production, mice were subjected to sublethal cecal ligation and puncture (CLP), an often used model for sepsis that recruits immature myeloid cells into the periphery [8]. Two days after CLP, CD11c-negative CD11b-positive cells were isolated from spleen and bone marrow for in vitro analysis. The CD11c^−^ CD11b^+^ splenocytes from septic TNFR2^−/−^ mice responded with lower iNOS mRNA expression and NO production to stimulation with LPS plus IFNγ compared to the respective cells from septic WT mice (Fig. 1C,D). Flow cytometric sorting of these splenocytes using the cell surface markers CD11b, CD11c, Ly6C, and Ly6G identified immature inflammatory myeloid cells (IMC) and more specifically monocytic myeloid-derived suppressor cells (CD11b^+^ Ly6C^high^Ly6G^−^, MO-MDSC) [9,10] as the functionally impaired TNFR2^−/−^ myeloid subpopulation with reduced NO production capacity (Fig. 1E).

To compare the suppressive capacity of MO-MDSC derived from WT and TNFR2^−/−^ mice, MO-MDSC and PMN-MDSC (CD11b^+^ Ly6C^int^ Ly6G^+^) were enriched from bone marrow and spleen of septic mice (Fig. 1F) and were tested for their suppressor activity of T cell proliferation after an overnight adherence period. Bone marrow MO-MDSC of septic mice suppressed T cell proliferation of both CD4 and CD8 T cells, being more suppressive for CD4 than for CD8 T cells (Fig. 1G–J), while spleen-derived MO-MDSC from septic mice were not suppressive (data not shown). The suppressive activity of bone marrow MO-MDSC of septic TNFR2^−/−^ mice was less strong compared to bone marrow MO-MDSC from septic WT mice. This reflected the reduced NO production capacity of CD11b^+^ splenocytes from septic TNFR2^−/−^ mice (Fig. 1D), which is considered as hallmark of suppressive MDSC [11]. Under these experimental conditions purified PMN-MDSC did not demonstrate marked NO production (Fig. 1E) nor were they suppressive for T cell proliferation (data not shown). These data demonstrate an impaired MDSC phenotype in TNFR2^−/−^ mice.

### Development of GM-CSF-derived MDSC from TNFR2^−/−^ mice

**Figure 2:**
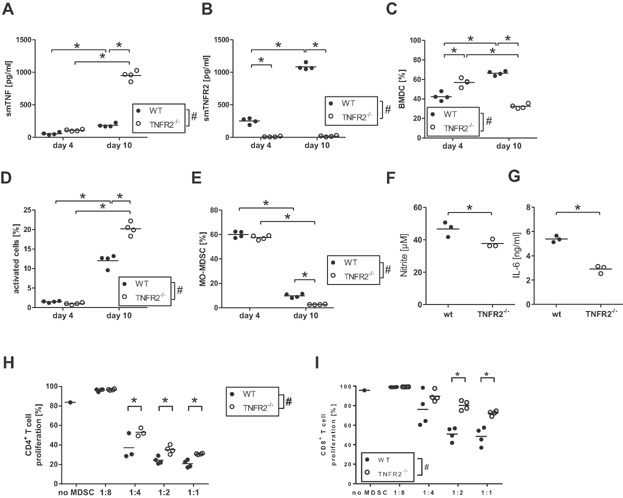
Differences between WT and TNFR2-deficient bone marrow-derived myeloid cells. (A,B) Bone marrow cells from WT (black circles) and TNFR2^−/−^ (white circles) mice were cultured in vitro for the indicated days and analyzed for soluble mouse TNF (A) and soluble mouse TNFR2 (B). Each symbol represents one mouse; the horizontal line represents the mean. ^#^ Indicates that two-way ANOVA demonstrated a significant influence (*P* < 0.05) of TNFR2 on the result, * indicates *P* < 0.05 in Bonferroni's post test. (C-G) Bone marrow cells from WT CD45.1 (black circles) and TNFR2^−/−^ CD45.2 (white circles) mice were co-cultured in vitro for the indicated days and analyzed for the relative population size (C), the levels of activated cells (D), and the levels of MO-MDSC (E) in the culture. Each symbol represents one mouse; the horizontal line represents the mean. ^#^ Indicates that two-way ANOVA demonstrated a significant influence (*P* < 0.05) of TNFR2 on the result, * indicates *P* < 0.05 in Bonferroni's post test. Cells were further separated by flow cytometric sorting according to the congenic markers CD45.1/2, re-stimulated with LPS plus IFNγ in vitro for 48 h and the levels of nitrite (F) and IL-6 (G) in supernatant were determined. In (F), each symbol represents an independent experiment; the horizontal line represents the mean. In (G), data from one experiment is shown – each symbol represents a technical replicate; the horizontal line represents the mean. * Indicates *P* < 0.05 in Student's *t*-test. (H,I) Bone marrow cells from WT (black circles) and TNFR2^−/−^ (white circles) mice were cultured in vitro and used in a T cell proliferation suppression assay. Each symbol represents one mouse; the horizontal line represents the mean. ^#^ Indicates that two-way ANOVA demonstrated a significant influence (*P* < 0.05) of TNFR2 on the result, * indicates *P* < 0.05 in Bonferroni's post test.

To test whether differentiation from precursor cells to mature myeloid cells might be altered in TNFR2^−/−^ mice, we followed the differentiation of bone marrow cells to dendritic cells (DC) from WT and TNFR2^−/−^ mice in the presence of GM-CSF as described earlier [12]. GM-CSF-derived myeloid cells represent the best in vitro culture system equivalent to DC arising in vivo during inflammation [13]. Also, development of MDSC has been observed in such cultures [14]. TNF was released into the supernatant during GM-CSF-driven differentiation of bone marrow cells (Fig. 2A). The TNF levels were higher in cultures derived from TNFR2^−/−^ compared to WT mice due to the absence of soluble TNFR2 in TNFR2^−/−^ cultures acting as TNF scavenger (Fig. 2B). To compare the development of DC derived from the two mouse lines under identical culture conditions, we co-cultured WT and TNFR2^−/−^ bone marrow cells at a 1:1 ratio. Congenic markers (CD45.1/2) were used for discrimination. On day 4, the proportion of TNFR2^−/−^ cells (CD45.2) in the cultures was higher compared to WT (CD45.1) cells but dropped strongly at later culture time (Fig. 2C). Proliferation of TNFR2^−/−^ DC was slightly reduced compared to WT DC as measured by BrdU-uptake on day 10 (data not shown). TNFR2^−/−^ DC had a higher percentage of activated cells (MHC-II^+^ CD80^+^ CD86^+^) (Fig. 2D) pointing to improved maturation of TNFR2^−/−^ DC. Together, these data show accelerated GM-CSF-driven differentiation and maturation of TNFR2^−/−^ bone marrow precursors to DC.

### Impaired MO-MDSC development in GM-CSF-driven bone marrow cell cultures from TNFR2^−/−^ mice

MO-MDSC, defined as CD11b^+^ Ly6C^+^ Ly6G^−^, in these co-cultures were analyzed in more detail. Until day 4 equal percentages of WT and TNFR2^−/−^ MO-MDSC were present in the cultures. Thereafter the fraction of TNFR2^−/−^ MO-MDSC decreased more quickly compared to WT MO-MDSC (Fig. 2E). After separation of WT and TNFR2^−/−^ DC by sorting according to the congenic markers, the capacity for production of NO (Fig. 2F) as well as of IL-6 (Fig. 2G) was tested, two important factors for suppressive MDSC [15,16]. GM-CSF-derived TNFR2^−/−^ DC produced less NO and IL-6 compared to WT DC. Corresponding results of reduced MDSC numbers and NO and IL-6 production capacity and increased maturation were seen in cultures of pure TNFR2^−/−^ cells compared to cultures of pure WT cells (data not shown). TNFR2^−/−^ cultures reproducibly yielded lower cell numbers (about 70%) after more than a week of culture. Since pure cell cultures from both mouse lines also contained equal quantities of MO-MDSC after 4 days, such day 4 cells were directly compared for suppressive activity in T cell proliferation assays. TNFR2^−/−^ MO-MDSC were less suppressive for proliferation of CD4 as well as CD8 T cells compared to the corresponding WT MO-MDSC (Fig. 2H,I).

To further differentiate the impact of soluble TNFR2 and membrane-bound TNFR2 on DC development, we added TNF inhibitors to in vitro cultures of WT, TNF-deficient, or TNFR2-deficient BM cells. Addition of TNF inhibitors (anti-TNF antibodies (V1q) or TNFR2-Fc construct (Etanercept, Pfizer, New York, NY, USA)) to bone marrow cell cultures significantly reduced the yield of DC in WT as well as in TNFR2^−/−^ cultures (Table1). A similarly significantly reduced yield of DC was found in cultures of cells from TNF-deficient mice without TNF inhibitors, indicating the requirement of TNFR1 activation for the generation of DC. Furthermore, the percentage of activated DC was significantly reduced in cultures of TNF-deficient cells and in cultures of WT and TNFR2-deficient cells containing TNF inhibitors, while under all these conditions significantly more activated DC were found in TNFR2^−/−^ cultures correlating with the enhanced TNF levels in the supernatant of such cultures. Following stimulation, the production capacities for NO and IL-6 differed markedly between WT, TNF-deficient, and TNFR2-deficient cells. Interestingly, while DC from cultures containing TNF-deficient cells produced significantly more NO than WT cultures, TNFR2^−/−^ DC had a significantly reduced production capacity of both NO and IL-6. As TNF inhibitors did not affect the levels of NO and IL-6 at all, this data also speaks for the importance of membrane TNFR2 activation for NO and IL-6 production.

**Table 1:** Comparison of GM-CSF-driven bone marrow-derived myeloid cells from WT, TNF-, and TNFR2-deficient mice

	CD11c^+^ (%)	MHC/II/CD86^+^ (%)	NO (μM)	IL-6 (ng/mL)
WT				
None	92.4 ± 0.1	12.7 ± 1.8	56.0 ± 1.9	12.2 ± 1.5
V1q	88.7 ± 2.0*	7.9 ± 0.9*	50.5 ± 0.1	10.7 ± 0.4
Etanercept	85.0 ± 0.3*	7.2 ± 0.9*	57.1 ± 4.1	11.5 ± 0.7
TNF^−/−^				
None	88.5 ± 0.4#	8.0 ± 0.4#	84.7 ± 2.4#	13.9 ± 1.7
V1q	87.9 ± 0.1	6.8 ± 0.2	82.3 ± 4.6#	11.7 ± 0.6
Etanercept	86.7 ± 0.4	6.6 ± 0.8	92.1 ± 3.4#	13.4 ± 0.0
TNFR2^−/−^				
None	89.2 ± 0.9	22.5 ± 0.9#	35.6 ± 0.3#	6.1 ± 1.0#
V1q	83.8 ± 0.1*^,^#	18.3 ± 0.0*^,^#	37.6 ± 1.1#	5.6 ± 0.6#
Etanercept	83.9 ± 1.6*	16.2 ± 0.4*^,^#	41.2 ± 1.0#	6.4 ± 0.3#

Data are mean and SD of technical duplicates and were analyzed with a one-way ANOVA followed by a Bonferroni post-hoc test.

* A statistically significant difference between the groups with either V1q or Etanercept and their respective control.

# A statistically significant difference between TNF-deficient or TNFR2-deficient mice and WT mice in the respective treatment group.

### Impaired MO-MDSC development in GM-CSF-driven bone marrow cell cultures from bone-marrow chimeras

**Figure 3:**
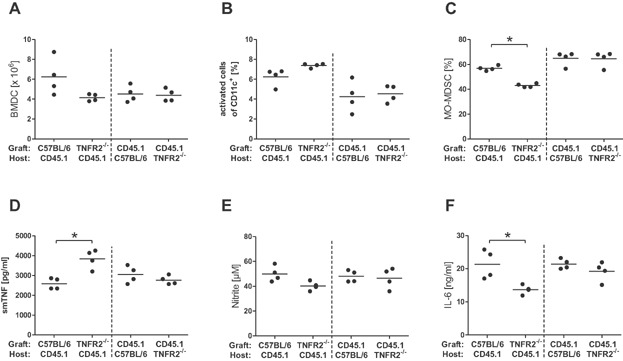
Differences in bone marrow-derived myeloid cells from WT or TNFR2-deficient chimeric mice. Bone marrow chimeras were generated by transferring WT CD45.2 or TNFR2^−/−^ CD45.2 bone marrow cells into irradiated WT CD45.1 host mice (left halves of the panels), or WT CD45.1 bone marrow cells into irradiated WT CD45.2 or TNFR2^−/−^ CD45.2 host mice (right halves of the panels). Following successful reconstitution, bone marrow cells were prepared from the chimeras and cultured in vitro in presence of GM-CSF. Cell yield (A), levels of activated DC (B), and levels of MO-MDSC (C) were analyzed on day 10 of culture. Cells were re-stimulated with LPS plus IFNγ and levels of soluble mouse TNF (D), nitrite (E) and IL-6 (F) in supernatant were assayed. Each symbol represents one mouse; the horizontal line represents the mean. Data were analyzed with a one-way ANOVA and * indicates *P* < 0.05 in Tukey's post test.

The presence of TNF and soluble TNFR2 did not affect the development of TNFR2-deficient cells in the bone marrow co-cultures. However, the lack of soluble TNFR2 might have already altered the precursor cells obtained from the bone marrow. To assess the impact of soluble TNFR2 on the bone marrow precursor cells, we generated four groups of chimeric mice by the adoptive transfer of bone marrow cells to irradiated host mice: WT C57BL/6 (identified by the allogeneic marker CD45.2) bone marrow into WT CD45.1 host mice (WT → WT), TNFR2^−/−^ bone marrow cells (CD45.2) into WT CD45.1 host mice (TNFR2^−/−^ → WT), and vice versa. More than 80% reconstitution of the hematopoietic system was achieved in the chimeric mice (data not shown). Following 8 weeks of reconstitution, bone marrow cells from the chimeric mice were cultured in the presence of GM-CSF to obtain DC. Supernatants of cultures from TNFR2^−/−^ → WT chimeras contained significantly less soluble TNFR2 compared to supernatants from WT → WT chimeras (*P* = 0.014; data not shown), indicating that most DC were descendants from the donor. In these TNFR2^−/−^ → WT chimeric cultures the yield of DC was lower compared to WT → WT cultures and the percentage of activated cells was higher though the differences were not significant (Fig. 3A,B, left half). The fraction of MO-MDSC in these TNFR2^−/−^ → WT cultures was significantly lower compared to the respective control WT → WT cultures, mirroring the results with TNFR2-deficient mice (Fig. 3C, left half). Stimulation of TNFR2^−/−^ → WT-derived DC induced higher TNF levels in the supernatant than stimulation of WT → WT DC, again demonstrating the good reconstitution (Fig. 3D, left half). In addition, lower NO and IL-6 production was induced in DC from TNFR2^−/−^ → WT chimeras, also repeating the results with TNFR2-deficient mice (Fig. 3E,F, left halves). Yield and proportion of activated DC was generally lower in chimeras with CD45.1 mice as donors but no difference was observed in any of the measured parameters in these chimeric cultures, independently of the recipient mouse line (Fig. 3A–F right half).

Together these data from GM-CSF-driven pure and co-cultures with and without TNF inhibitors and from TNFR2^−/−^ chimeras indicate that the reduction of MDSC function is based on deficiency of membrane TNFR2 and is independent of the culture environment.

## Discussion

Experimental evidence has been growing for an independent role of TNFR2 in immunological and inflammatory reactions [2]. In particular, TNFR2 has been claimed to play an important role by generation of Treg [5,17,18] and MDSC [7,6]. Previously the requirement of TNFR2 expression on non-T and non-B cells for NO production and for suppression of tumor growth has been described [19]. Immature myeloid cells with characteristics of MDSC have been found to be an important source of TNF-dependent NO production [20]. Together these findings point to a critical role for TNFR2 expression on myeloid cells for proper MDSC function. The observations described in this study of impaired capacities of MDSC from TNFR2^−/−^ mice for NO and IL-6 production and for suppression extend these previous reports and constitute a so far non-described phenotype of TNFR2^−/−^ mice. High expression levels of iNOS are typical for MDSC (as reviewed by Gabrilovich et al. [21]). The suppressive effect has been explained by down-regulation of the zeta-chain of the TCR, by interference with the IL-2 receptor signaling and by desensitization of the TCR. IL6 has been shown to promote MDSC mobilization and accumulation in the tissue via gp130-STAT3 [16].

Furthermore, we determined whether direct activation and signaling of TNFR2 is required for optimal function of MO-MDSC or whether the TNF neutralizing capacity of soluble TNFR2 contributes to the MDSC phenotype. Our data clearly demonstrate that for the in vitro differentiation the level of soluble TNF in the medium and, therefore, also soluble TNFR2 as TNF scavenger has no effect on the function of TNFR2-deficient MDSC. Thus, the results published by Sade-Feldman et al. [6] demonstrating a TNF-dependent retardation effect on the differentiation of MO-MDSC were nicely reproduced in our GM-CSF-containing co-culture experiments. In addition, our data extend these findings by demonstrating that the requirement for membrane TNFR2 on the myeloid cells is critical while the available levels of biologically active TNF in the in vitro culture environment are irrelevant. These findings are supported by the data obtained with TNF inhibitors during the GM-CSF culture of bone marrow cells, where TNFR2-deficient cells consistently demonstrated higher activation levels and lower NO and IL-6 production levels than WT cells. This phenotype was largely independent of the presence of TNF inhibitors and, therefore, also independent of the activation of the TNFR1. The observation of higher NO production capacity in cultures with TNF-deficient myeloid cells clearly speaks for opposite effects of the two TNF receptors on MDSC functions, however, the understanding of the mechanisms in detail remains elusive. The observation of the maintained phenotype of TNFR2-deficient MDSC in cells experiencing the same milieu in vitro strongly argues for a cell autonomous signaling role of TNFR2. Still, epigenetic modifications cannot be excluded as cause for the TNFR2^−/−^ MDSC phenotype. Furthermore, the receptor crosstalk between TNFR1 and TNFR2 guaranteeing the delicate balance of TNF-dependent signaling [2] might be affected in TNFR2^−/−^ mice.

As TNFR2-deficient mice are more susceptible for pathological symptoms in different mouse models, the hypothesis that TNFR2-signaling is protective emerged in the field. Speaking against this conclusion that the lack of TNFR2 signaling explains all effects seen in TNFR2^−/−^ mice, we recently published the finding that the reduced capacity for IL-12 production of splenic myeloid cells from TNR2^−/−^ mice is due to habituation after being repeatedly exposed to enhanced levels of endogenous TNF due to the lack of soluble TNFR2 [22]. However, in contrast to the altered capacity of TNFR2-deficient dendritic cells for IL-12 production, in this study we show that regulated differentiation and function of MDSC, indeed, requires TNFR2 in its form of a cell membrane-spanning receptor by demonstrating that the lack of membrane-TNFR2 affects MDSC generation and function in vitro and in vivo independently of soluble TNFR2. The here described impaired immune regulatory capacity of TNFR2-deficient MDSC might contribute to the reported higher susceptibility of TNFR2-deficient mice to pathophysiological consequences of sepsis [23], to impaired tumor growth [24], to failure of immunoparalysis after sublethal sepsis [25], and to the reported protection from sepsis-induced mortality by LPS pre-treatment [26,27].

## Materials and Methods

### Animals

C57BL/6 mice were purchased from Janvier (Le Genest, France). TNFR2-deficient mice (C57BL/6-Tnfrsf1b^tm1Mwm^) [28] were purchased from The Jackson Laboratory (Bar Harbour, Maine, USA) and CD45.1 (C57BL/6J Ly5.1) [29] from Charles River (Sulzfeld, Germany). TNF-deficient mice [30] were bred in the animal facility of the University of Regensburg. Deficiency of TNFR2 expression was verified by PCR and lack of soluble TNFR2 in urine by ELISA. Mice were housed in the animal facility of the University of Regensburg and handled in accordance with institutional guidelines (Az:54-2532.1-32/08, 27/10).

### Myeloid cell stimulation

CD11b-positive cells depleted of CD11c^+^ were isolated by MACS separation from splenic single cell suspensions following the manufacturer's protocol (Miltenyi Biotec, Bergisch Gladbach, Germany). Peritoneal exudate cells (PEC) were harvested 16 h after injection of 1 mL PBS ip by peritoneal lavage. For generation of bone marrow-derived dendritic cells (BMDC), mice were killed and femura and tibiae were dissected, placed shortly in 70% EtOH, both ends were cut, and bone marrow was flushed out with PBS. Cells were washed once with medium (RPMI 1640 with 10% FCS, Pen/Strep, 50 µM 2-ME) and resuspended in medium containing granulocyte–macrophage (GM)-CSF for generation of BMDC as described previously [12]. For some experiments, TNF inhibitors (Etanercept; Pfizer Limited, Kent, UK) or V1q [31] were added to the cultures at 10 µg/mL or 2 µg/mL, respectively. PEC or BMDC on d4 or d10 of culture (2.5 × 10^5^/mL) were stimulated in medium at 37°C with LPS (125 ng/mL, *S. abortus equi*, kindly provided by M. Freudenberg) and mouse IFNγ (50 ng/mL, Peprotech, Hamburg, Germany) for 48 h and used for isolation of RNA or supernatants for determination of cytokines by ELISA or NO. ELISA kits (R&D Systems, Wiesbaden, Germany) were used for quantification of mouse TNF, IL-6 and soluble TNFR2 in supernatants. Nitrite concentrations were determined using Griess reagent measuring the optical density at 540 nm.

### T cell suppression assay

CD11b^+^ cells were isolated from spleen and bone marrow cells (prepared as described above) by magnetic separation with a negative selection antibody cocktail according to the manufacturer's instructions (Miltenyi Biotec). Next, Ly6G^+^ cells were separated by positive selection with magnetic beads. The resulting positive population was CD11b^+^ Ly6C^int^ Ly6G^+^ (PMN-MDSC). The negative population was mainly CD11b^+^ Ly6C^+^ Ly6G^−^ (MO-MDSC) with some contaminating PMN-MDSC. Various numbers of cells were placed in 100 µL cultures overnight before addition of 100 µL culture medium with 2 × 10^5^ 5,6-carboxyfluorescein succinimidyl ester (CFSE)-labeled splenocytes as responders. Cells were stimulated for 3 days by anti-CD3e-antibody (1 µg/mL) and anti-CD28-antibody (0.5 µg/mL) and analyzed by flow cytometry. In other experiments, bone marrow cells were cultured with GM-CSF as above for 4 days and used as suppressive cells. Proliferating T cells were calculated by gating the respective T cell subpopulation and quantifying the percentage of T cells with diluted CFSE content.

### Cecal ligation and puncture (CLP)

Mice were anesthetized (75 mg/kg Ketamin (Parke, Davis & Company, Munich, Germany) and 16 mg/kg Xylazin (Bayer AG, Leverkusen, Germany)) ip. The cecum was exteriorized and 30% of the distal end was ligated and punctured once with a 27G needle to achieve a sublethal CLP [32].

### Generation of bone-marrow chimeras

Host mice were irradiated two times with 5 Gray from a linear accelerator. After irradiation host mice were anesthetized and 5 × 10^6^ bone marrow cells prepared from donor mice as described above were injected i.v. After 3 weeks, reconstitution was checked using venous blood samples from every mouse. After 8 weeks of reconstitution the mice with the highest degree of reconstitution were chosen for the experiments.

### Flow cytometry

Following lysis of red blood cells in single cell suspensions from spleens and bone marrow, cells were incubated with antibodies against CD16/32 to block non-specific antibody binding and stained with fluorochrome-labeled antibodies. Fluorochrome- or biotin-labeled antibodies detecting mouse CD11c, CD11b, CD4, CD8a, B220, Ly6C, Ly6G, CD86, CD80, and MHC-II as well as fluorochrome-labeled streptavidin were purchased from BD Biosciences (Heidelberg, Germany), eBioscience (Frankfurt, Germany), Miltenyi Biotec, or Invitrogen (Darmstadt, Germany). Cells were measured on a BD LSR-II or a BD FACSCalibur flow cytometer and analyzed using the FacsDiva or CellQuest softwares (BD Biosciences). For sorting of cell populations, antibody-labeled cells were sorted on the FACS Aria cell sorter (BD Biosciences). After sorting, cells were washed twice with medium and used for the respective cellular assay. Sorted cells were reanalyzed on the BD LSRII and showed purities higher than 98%.

### Quantitative real-time PCR

RNA was prepared from samples using the Nucleospin RNA II kit according to the manufacturer's instruction (Macherey-Nagel, Düren, Germany) and cDNA was generated by transcription with MMLV (Promega, Mannheim, Germany). Primers for iNOS mRNA (fw: gct gtt ctc agc cca aca at, rv: tgc aag tga aat ccg atg tg) and β-actin (fw: tca ccc aca ctg tgc cca tct acg a, rv: gga tgc cac agg att cca tac cca) were purchased from Metabion (Martinsried, Germany). The real-time PCR was performed with the iQ SYBR Green Super Mix (BioRad, Munich, Germany) on an iQ5 iCycler (Biorad) with 45 cycles (20 sec 95°C, 60 sec 59°C). The expression levels of iNOS were determined as relative expression compared to β-actin by the Δ*C*_t_ method.

### Statistics

Student's *t*-test, one-way analysis of variance (ANOVA) with Tukey or Bonferroni post-hoc test or two-way ANOVA with Bonferroni post-hoc test were used in experiments with two or more experimental groups. *P* < 0.05 was accepted as significantly different. All statistics were performed using GraphPad Prism 5.0 (GraphPad Software Inc., La Jolla, USA).
